# Understanding Another Person's Emotions—An Interdisciplinary Research Approach

**DOI:** 10.3389/fpsyt.2018.00414

**Published:** 2018-09-06

**Authors:** Georg Juckel, Christine Heinisch, Anna Welpinghus, Martin Brüne

**Affiliations:** ^1^Department of Psychiatry, Ruhr University Bochum, LWL University Hospital, Bochum, Germany; ^2^Institute of Philosophy II, Ruhr University Bochum, Bochum, Germany

**Keywords:** emotion, emotion recognition, expression of emotions, social context, interaction theory

## Abstract

An interdisciplinary research perspective is developed concerning the question of how we understand others' emotions and how reliable our judgment about others' emotion can be. After an outline of the theoretical background of emotions, we briefly discuss the importance of prior experiences and context information for the recognition of emotions. To clarify this role, we describe a study design, utilizing emotional expressions and context information while controlling for prior experiences and the actual emotional situation to systematically approach these questions.

## Emotion recognition as pattern recognition

Darwin's work on the expressions of emotions in humans and animals can be regarded as a milestone in emotion research (1). He and others like Wilhelm Wundt in Germany focused on innate and inherited aspects of the expressions of emotions and their universality across cultures (2) see also (3, 4). Darwin and Wundt were well aware of the problem that emotion recognition is also dependent on individual experience, at least with regard to more complex emotional states. More recently, the problem of matching expression and recognition (or comprehension) has fueled philosophical debates about how we understand others in the absence of direct access to their feelings or thoughts—known as the “problem of other minds.” This problem comes in two variations: conceptual and epistemological. The conceptual problem is concerned with the question of whether and how we can have reliable conceptualizations and knowledge of others' mental states. The epistemological problem is concerned with the skeptical question of whether we can have access to another person's mental states at all. Leaving philosophical skepticism behind, we want to focus on the first question, because a convincing story about how we manage to recognize others' emotions is sufficient to ignore the radical skepticism. For emotions, as we will argue, access to other minds can be convincingly described and empirically disentangled. Independent from any specific theory of emotions, we can rely on the following argument: First, theories of emotion which do not identify emotions with inner feelings accept that emotions are not purely private mental entities but at least have “public” components (e.g., facial expressions and behavioral tendencies) which are clearly observable from an outside perspective. Second, for theories that identify emotions with inner feelings and claim that emotions are private, we still have access to these inner feelings of another person by sharing an emotional situation with the other (both being attacked by an aggressive dog) or by utilizing means of communication including verbal report.

It is important to distinguish two ways in which we can understand others' emotions. One can either develop a *cognitive understanding*, if one knows which emotion concept is appropriate to explain and predict the expression and behavior one observes. Or one can know how it feels like to be in the others' affective state, or, in other words, develop an empathic understanding. Ideally, we can rely on both types of understanding, but they are independent of each other. This can be illustrated by the profile of psychopathy, which typically includes high competence in cognitive understanding but a lack of empathic understanding. A similar situation with only cognitive understanding can also result from a normal ontogenetic situation, for example, if a child has never experienced the death of a person she is personally attached to, but learns that this happened to a friend and that he is in deep grief; it may be that children, until they have other deeply sad experiences to relate them to this phenomenon, still only develop a cognitive understanding of grief, by getting familiar with the emotional pattern typical for grief. Still, they probably do not know how it *feels* like to be in deep grief. In the other direction, we may mention the case of people suffering from down-syndrome who are often sensitive in developing an empathic understanding by registering whether the other is feeling well or not. But they lack a cognitive understanding of the complex situation and the related complex emotion that includes the positive or negative feelings of the other. In this context, the important differentiation of cognitive and affective parts of the theory of mind should be noted (5). Ideally, we would develop experiments which distinguish between cognitive and empathic understanding. In the first approach described here, we will not be able to do this. We instead focus on everyday cases of recognizing emotions, which is a combination of empathic and cognitive understanding. Systematic disentanglement is an important aspect to include in all future studies of emotions.

We would like to discuss and investigate the conditions which support or distort emotion recognition. One question is to what extent one's own prior experience of a specific emotion enables emotion recognition, and another is to which extent one's actual emotion in the situation (e.g., a background mood), influences emotion recognition. For example, it is known that patients with borderline personality disorder or posttraumatic stress disorder, who have experienced torture or pain, are more experienced in recognizing fear in other people (6).

Our background theory of emotion is the pattern theory of emotion (7) according to which emotions consist of a pattern of characteristic features. Thus, they can be described as phenomena comprising a range of aspects from typical physiological reactions, behavioral dispositions over specific facial and bodily expressions, and the typical phenomenal experience to characteristic cognitive attitudes or cognitive modulations such as shifts of attention. In an emotional episode the activated typical features are integrated with an intentional object into an appraisal of the situation with this intentional object. For each emotion (we are speaking of emotional episodes only), we can identify a typical pattern of characteristic features. For example, in the case of fear this includes increased heart-rate, wide open eyes, a disposition to flight or freeze, vigilance to threat, a typical facial expression, and body posture as well as the typical experience of fear, and it may involve a typical cognitive evaluation of, for example, the attacking dog as a dangerous pit-bull. As we argued elsewhere (7), none of these features is necessary (except typical physiological arousal) or sufficient for fear. To implement an episode of fear, we only need an integration of a minimal number of features with an intentional object (8), which allows for much variation of features realized in a situation. Furthermore, the relevant features are not totally independent from one another: there are various causal, constitutive, and conceptual links between them. If we presuppose—as we do—that emotions are individuated as patterns of characteristic features, then emotion recognition can be described as a form of pattern recognition. By itself, the concept of pattern recognition is not new. Recognition of emotional facial expressions has often been described as pattern classification in humans and in machines (1, 4). Facial expressions are taken as natural signs for especially basic emotions (9). Facial expressions of basic emotions are relatively easy to label and often it is sufficient to see only parts of this face to receive a correct activation of the relevant pattern and thereby to have a correct perception of the emotional state. However, when it comes to more complex emotions, classifying emotions based on facial expressions alone will not work (because it remains underdetermined). We have developed the idea of pattern recognition for complex emotions as well. We can account for test anxiety as anxiety that also involves typical cognitive attitudes like thinking that the test is decisive for one's career and very difficult to pass. This means that the pattern we aim to recognize does not only consist of visual features as in the case of facial perception (10). Recognition of emotions consists of the recognition of complex patterns which include easily visible as well as non-visible features like the phenomenal experience and the cognitive attitudes which are typical for test anxiety. To account for these cases of emotion recognition, we distinguish three cases of recognition where the first two are variants of emotion recognition by perception and only the third variant goes beyond perception (11). We distinguish (1) (a basic form of) perceiving an emotion in the (near) absence of any top-down processes, and (2) perceiving an emotion in a way that significantly involves some top-down processes (a form of perception strongly modified by background knowledge, for example). Both types of emotion perception can be distinguished from (3) an inference-based evaluation of an emotion pattern. The latter presupposes a stable evaluation of an emotion as being F, which then may be modified or re-evaluated by reflecting on the information. The principle place to account for the role of contextual information in emotion recognition is clearly going beyond the first strategy of direct perception without any top-down processes (i.e., no background information is shaping the perceptual recognition). We will leave it open in this article whether the role of contextual information is best described as a case of strategy 2 (i.e., rich perceptual experience) or strategy 3 (inference-based interpretation). Our focus here is to discuss and outline an experiment to clarify the role of contextual information as well as prior experiences of an individual in emotion recognition.

## Emotions in context

Usually emotions are not observed in isolation—one sees the other embedded in an environment that contributes additional contextual information (12) see also (13). Thus, the environment provides a frame for the perceptual recognition or the inference-based interpretation of emotional responses: when a person observes another in a situation that the observer renders dangerous, he or she will likely attribute fear to the observed subject. Some pragmatic situations partly derive their meaning from social conventions. If a person is familiar with these, s/he routinely uses them to understand others' emotions. There is evidence in the literature indicating that social contexts play an important role in emotion recognition. For example, Carrol and Russell (14) show that a semantic background story of a restaurant invitation can modify emotion recognition even when dealing with a typical Ekman face of a basic emotion like fear, anger, sadness, or disgust. The restaurant story to be read by the participants clearly indicates that the relevant person is angry due to unfair treatment. After reading the story, participants immediately see a typical Ekman face of fear; due to the influence of the story, the evaluation is significantly shifted from fear to anger [for a discussion see (15)]. Thus, we aim to further investigate the role of contextual information in emotion recognition. To best mirror everyday situations, we will not use semantic stories, but situate a facial expression within a natural visual scene.

Before presenting our framework of investigation, we would like to situate the role of contextual information in our theory of the nature of emotion as pattern of characteristic features. We previously described the typical constitutive features of fear. Contextual factors were not mentioned, and we think that they should be seen as mere pragmatic features modulating an affective state, not as being constitutive. For example, it is possible to experience test anxiety just by thinking about an upcoming exam even if the context is a relaxed bar situation with some friends. An analysis of this situation illustrates that test anxiety is an affective state for which the cognitive evaluation is constitutive (e.g., “The exam is coming up next week. It is very important for my future. It will be very difficult, and I will likely fail”). For being in such an affective state and having such an emotion, the context is important, but not constitutive. This is radically different for emotion *recognition*: To activate a typical pattern of an emotion, the typical context is essential for the observer. Contextual factors include most prominently (a) the pragmatic context in which the emotion occurs and (b) knowledge of the person having the emotion. As argued, these factors do not belong to the aspects of emotion individuation: Fear is fear, no matter whether an anxious person or a courageous person is afraid. It remains fear no matter whether it occurs in an obviously dangerous setting or not. However, fear in another person is much easier to recognize in a setting that assigns danger to the observer than it is in a (seemingly) safe setting. The fact that people make use of such contextual information does not mean that the pragmatic situation or aspects of the bearer's history or personality are part of the emotion, but these aspects are essential for emotion recognition. Wallbott et al. (12) distinguished between contextual information from within a behavioral category (e.g., different components of a facial expression), accompanying cues such as gestures and body posture, and situational factors (we prefer to talk about pragmatic context information), all of which can occur simultaneously or contingent, that is, distinct from the “basic” expression in space and time (12). Our aim is to investigate the role of pragmatic context information for the pattern recognition of emotions. To do this, we followed in the footsteps of literature reporting the effects of visual contextual information on recognition of facial expression in healthy and psychiatric cohorts (16, 17).

## Natural emotion recognition: the roles of context, mood, and prior experiences

Recognizing stereotypical faces is a relatively easy task and only simple pattern recognition skills are required. In everyday life, however, we usually meet people displaying ambiguous/plurivalent facial expressions often changing over short time-periods. In everyday situations, contextual information is needed to activate the correct pattern of the emotion that the observed person has. How do we make use of contextual information? We have designed a study providing a more natural facial recognition task than some of the classic tasks. Our material comprises photos depicting real life situations like festivals, sport events, and vacations. The faces of the protagonists involved were prepared to present ambiguous expressions such that without contextual information, the emotions are rated differently by several raters as showing happy, disgusting, fearful, angry, surprised, or sad expressions. Moreover, the faces are then presented embedded in different backgrounds so that the influence of context can be examined. Contextual information seems to elicit a widely shared impression that a person in this context has one specific emotion, such as feeling sad, happy, neutral, angry, or fearful. Only when the context explicitly does not fit the emotions displayed in the facial expression is contextual information ignored. Wallbott et al. (12) reported that when people predict positive emotions the information conveyed by a person (by means of facial expression, for example) has greater weight compared to contextual factors. In contrast, when predicting negative emotions, contextual information gains in importance relative to personal information. Moreover, current mood seems to influence one's judgment of another's emotional state. One part of our hypothesis is that ambiguous faces which are misinterpreted when shown in isolation become intelligible when viewed in the context of a given situation (18, 19) see also (20), which indicates an important role of context in emotion recognition. In our study, we follow a more natural design by showing ambiguous facial expressions, which are rated differently by several individuals who only perceive the facial expressions in isolation. In addition, each facial expression is put into different contexts so that the context influences the perception of the face and may bias our participants to perceive a certain emotion. By using this paradigm, we can investigate the participants' strategy of how to evaluate facial expressions on a perceptual basis. This part of the study probes how much information about an emotional pattern the observer needs to feel certain concerning the evaluation of the others' emotions.

A second hypothesis to be tested is the influence of the participants' own emotional situation (we focus on the observers' mood) and their own prior experiences. Both features are relevant for emotion recognition in addition to contextual information. The participants' own emotional situation and their own experiences with life events eliciting strong emotions are reported. For instance, a depressive person needs happier facial expressions to label them correctly (21, 22). Moreover, evaluations are easier/faster, when one's own mood and contextual information, as well as parts of the emotional pattern in the face, are congruent (23). When considering the role of one's own internal states, we must account for the effect of the mirror neuron system (MNS) in understanding others. Iacoboni (24) describes the MNS as pre-reflective, automatic mechanism of mirroring which consists of the activation of the same neurons when doing an action and when observing it. For example, this mirroring role of specific neurons has been shown for disgust, such that the same neurons activate when experiencing disgust and when perceiving it in the face of another person (25). Because the mirror neuron activation is automatic and does not normally induce a *conscious experience* of the emotion of the other but only an unconscious representation, the role of the MNS for emotion recognition is intensely debated (26, 27). The standard interpretation of the MNS-camp concerning social cognition is that the MNS produces the same emotional state in the observer as in the observed person and that this state—despite being unconscious—is the central basis for projecting this internal state to the observed person by attributing the correct emotion. This process is described as low-level simulation by Goldman (28). Low-level simulation is a subpersonal process initiating the projection of one's own registered state onto the other. This view is supported by the observation that muscle activity is related to activation of the MNS in the brain when seeing emotional faces [(29); for further evidence see (28)]. But on the other hand, the activation of the MNS remains unconscious and often the observer's conscious emotional situation is different from the other's. Even if the observer is emotionally involved in a different project (e.g., angry about a job), this does not prevent him/her from observing the other's emotion (e.g., of being sad). Further criticism is developed in Gallagher (30) and Newen and Schlicht (31). For the purpose of this article, we accept that the MNS contributes to emotion recognition, but it is not sufficient to explain the result of the perception or evaluation because the perceptual process of seeing an emotion needs more determinants and can allow for even rich contents to interfere (32).

Because our main focus of discussing the MNS is the question of whether it plays a crucial role in determining the involvement of one's own emotional state in emotion recognition, the evidence is mixed and the role of the MNS is overstated by the MNS-camp, claiming that mirror neuron activation is one of the components of understanding others including emotion recognition. We may add to other evidence pointing in different directions: on the one hand, it is reported by Adolphs et al. (33) that an individual who suffered from bilateral destruction of her amygdalae—widely recognized to play a central role in mediating fear—showed severe impairments in both her experience of fear and in face-based recognition of fear. On the other hand, we have evidence from investigating psychopaths that this group of people have a high sensitivity and reliability in recognizing fear in others while they have a strongly flattened level of experiencing emotions, including fear. According to our view, these data can be reconciled by accounting for several independent and parallel mechanisms of emotion recognition [this multiplicity view is developed in Newen (11).

Furthermore, the result of a variety of mechanisms may be two different types of understanding which we highlighted at the beginning of the article: empathic understanding and cognitive understanding.

Evaluating the other's mental state is often more reliable if it is not combined with equating it with one's own emotional state—the idea of decoupling. The better people are at keeping their own feelings (current feelings as well as history) apart from the other's, the better they are at recognizing other people's mental states.

Empathic understanding of the other's emotion remains easier in cases when one has the same emotion. Cognitive understanding of the other's emotion is easier if one does not undergo the same emotion but is in a neutral emotional state. With questionnaires, we will assess the actual mood of our participants at moment of testing, which could have an influence on assessing the emotions of others.

The combination of the perceptual task with ambiguous faces in different contexts and the questionnaires assessing the participant's own mental state will contribute to the research question of whether we use knowledge about the situation or of the facial expression to ascribe emotions to others. If participants heavily rely on contextual information, this would pose a challenge for embodied or low-level simulation theories (especially the MNS theories of social cognition) because for these theories, the mechanism for recognizing emotions is the simulation of the other's state based on his/her facial and bodily expression modulated by mirror neuron activation. It is conceivable that different subjects use different strategies. However, we want to investigate the role of contextual information and one's own mood for emotion recognition since this has not been tested so far in a combined design, while we focus on pattern recognition as main mechanism for recognizing the emotions of others (see mechanism 3 above).

## Study design for an interdisciplinary research project

**Figure 1:**
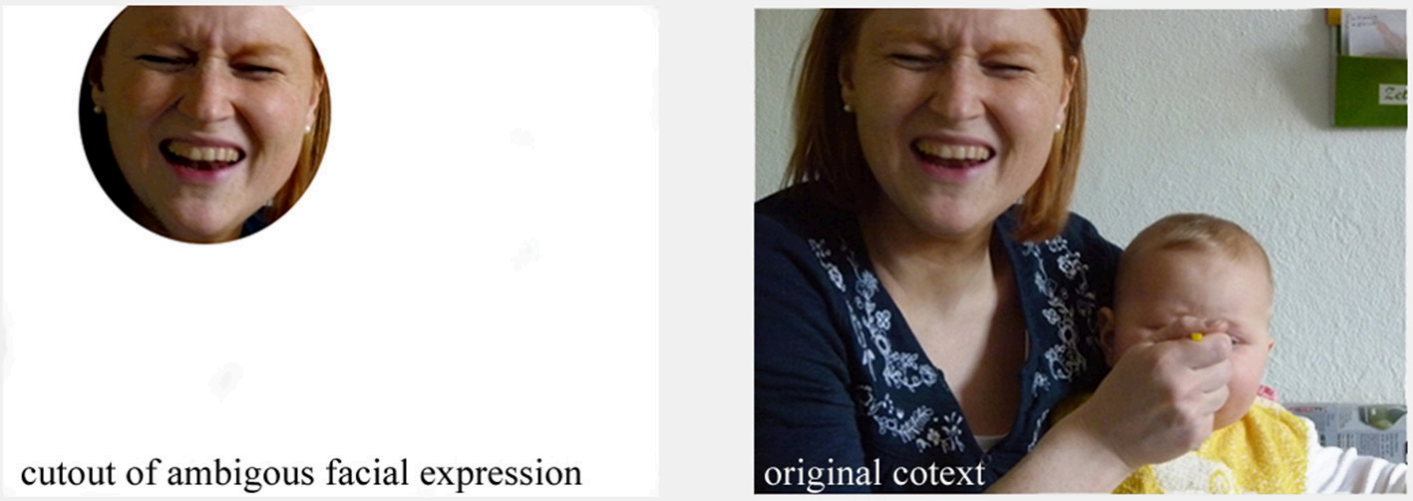
Cutout of ambiguous facial expression which has been rated to some degree sad (38%), fearful (48%), disgusted (32%), happy (36%), and aggressive (25%) on a scale from 1 to 100, where 1 is “does not show this emotion at all” and 100 is “definitely the named emotion.” The context shows a daily situation of a mother feeding her child. It is likely that she is disgusted by and laughing about how her child eats. (Figure copyright Dr. C. Heinisch, private photos with permission and written consent for publication by the imaged person).

**Figure 2:**
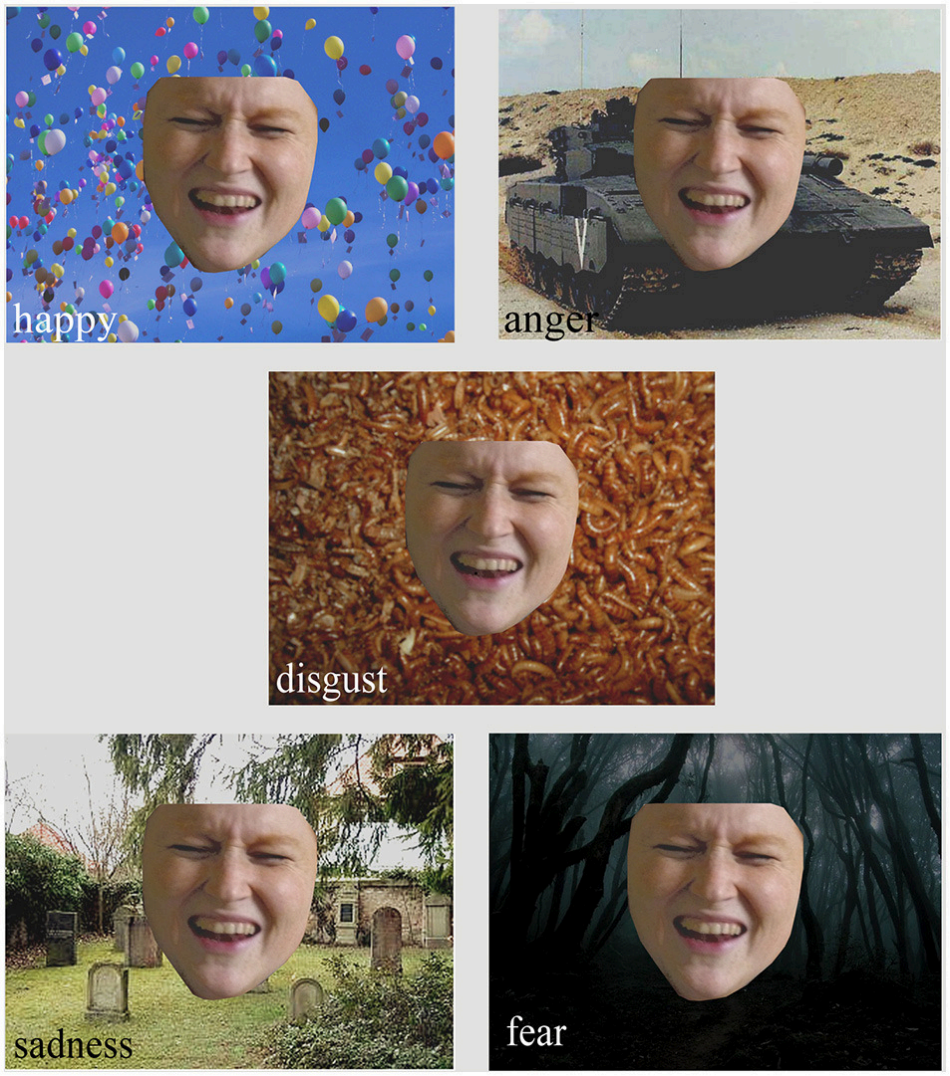
The same cutout facial expression put into different contexts. All backgrounds have been rated as higher than 65% in the named emotion. It is assumed that empathizing with the face in different contexts can change the observer's judgement of what the person in the picture is feeling. (Figure Copyright Dr. C. Heinisch, private photos with permission and written consent for publication by the imaged person).

We searched the Internet and private databases to find pictures of faces with ambiguous emotional expressions. After pre-testing, we chose nine pictures with a high standard deviation, indicating no clear emotional state, for further study preparation. The face was cut out of the original context and pasted over a pre-rated emotional background (e.g., a cemetery, balloons, worms, see Figures 1, 2) so that the contrasts of background and faces of all combinations were matched according to the means of the pilot ratings. Our participants (psychologically healthy individuals and people with psychiatric disorders) will view these pictures on a computer screen and be asked to label the facial expression as happy, sad, fearful, angry, surprised, or disgusted. In this study, participants will be asked to feel with the person, without any time limitation, but with the instruction of giving short and intuitive answers, and then to choose the label which comes closest to their impression of the emotion of the other. Afterwards, the participants will be asked how certain they feel about their answer. In the following questionnaire, the face will be presented in the original context and after rating the facial expression again, the participant will be asked what served as an indicator of the emotion: the facial expression, the eyes, or the context. By using this experimental design, we can compare whether and when subjects changed their minds about the facial expression of the person in the picture. The participants can either change their opinion with different contextual information or they can keep their initial opinion. The result will enable us to clarify the role of contextual information in emotion recognition. Our working hypothesis is that contextual information is strongly integrated into the process of emotion recognition in everyday situations. If this turns out to be true, then one explanation for this is that perceptual processes of emotion recognition can be cognitively penetrated by the contextual information such that the observable input features (face, body posture, behavior, etc.) are organized to form a unified perceptual experience of the others' emotion in the light of contextual information (32).

To test whether participants' present mood interferes with their tendency to label the face, a questionnaire will be given asking participants to report their own mood by marking different adjectives. These aspects of mental state can be assigned to six higher dimensions: performance-related activity, general inactivity, extra/introversion, general well-being, emotional temper, and fear. Temperament as personal marker will be measured by the Temperament and Character Inventory [TCI, (34)], which measures curiosity, harm avoidance, dependency on reward, and stickiness. The ability to experience one's own emotions will be measured with another questionnaire [see (35)], which is divided into seven scales: (1) acceptance of one's own emotions, (2) experience of emotional overflow, (3) experience of absence of emotions, (4) body-related symbolization of emotions, (5) imaginative symbolization of emotions, (6) experience of emotion regulation, and (7) experience of self-control. These scales measure how the participants experience their own feelings, evaluate them, and deal with them. Finally, empathy will be measured with the Interpersonal Reactivity Index [IRI, (36)], which comprises four subscales of perspective-taking, fantasy, empathic interest, and personal suffering. By using these questionnaires, we aim to identify critical aspects which allow us to recognize the emotions of someone else. Namely, how does mood, personal experiences, or ability to empathize and experience emotions influence emotion recognition and our feeling of certainty about our evaluation?

As a pilot study, healthy subjects will be tested and then will be compared to a psychopathological group suffering from difficulties with inferring other people's mental states. For instance, patients with schizophrenia are impaired in both emotion perception and contextual processing (37, 38). There is only one study showing a reduced influence of contextual information upon judgments of emotional intensity from facial expressions in schizophrenia (39), but there are also findings which report similar influence for patients with schizophrenia (40). However, it remains unclear how much of one's own biographic information these patients use to label emotional expressions, because self-processing and self-recognition are known to be impaired in schizophrenia patients (41). Although they decouple their own mood and experiences from those of others, they may not be able to use this personal background information for better emotion recognition. A study design including emotional expressions, context information, and self-reference, as presented here, is therefore needed to clarify the impact of these variables on emotion perception in schizophrenia. This knowledge is crucial to improving therapeutic approaches based on social cognition because emotion perception is crucial for successful social interaction.

## Conclusion

We here provide an interdisciplinary research perspective combining a philosophical theory of emotions (i.e., the pattern theory of emotions), with recent developments in psychology and psychiatry concerning our understanding of another person's emotions. We outlined a theoretical background for the importance of contextual information in the recognition of emotions. We then described a study design comprising emotional expressions and contextual information, in which we will empirically investigate the role of context in emotion recognition with the working hypothesis that context can sometimes be an essential component in the process of emotion recognition and not just a modulating factor.

## Author contributions

All authors listed have made a substantial, direct and intellectual contribution to the work, and approved it for publication.

### Conflict of interest statement

The authors declare that the research was conducted in the absence of any commercial or financial relationships that could be construed as a potential conflict of interest.
